# Fine Tuning of Tissues' Viscosity and Surface Tension through Contractility Suggests a New Role for α-Catenin

**DOI:** 10.1371/journal.pone.0052554

**Published:** 2013-02-04

**Authors:** Tomita Vasilica Stirbat, Abbas Mgharbel, Selena Bodennec, Karine Ferri, Hichem C. Mertani, Jean-Paul Rieu, Hélène Delanoë-Ayari

**Affiliations:** 1 Institut Lumière Matière, UMR5306 Université Lyon 1-CNRS, Université de Lyon, Villeurbanne, Lyon, France; 2 Université de Lyon, Université Lyon 1, INSERM U1052, CNRS UMR 5286, Centre de Recherche en Cancérologie de Lyon, Centre Léon Bérard, Lyon, France; Institute of Science and Technology Austria, Austria

## Abstract

What governs tissue organization and movement? If molecular and genetic approaches are able to give some answers on these issues, more and more works are now giving a real importance to mechanics as a key component eventually triggering further signaling events. We chose embryonic cell aggregates as model systems for tissue organization and movement in order to investigate the origin of some mechanical constraints arising from cells organization. Steinberg et al. proposed a long time ago an analogy between liquids and tissues and showed that indeed tissues possess a measurable tissue surface tension and viscosity. We question here the molecular origin of these parameters and give a quantitative measurement of adhesion versus contractility in the framework of the differential interfacial tension hypothesis. Accompanying surface tension measurements by angle measurements (at vertexes of cell-cell contacts) at the cell/medium interface, we are able to extract the full parameters of this model: cortical tensions and adhesion energy. We show that a tunable surface tension and viscosity can be achieved easily through the control of cell-cell contractility compared to cell-medium one. Moreover we show that 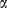-catenin is crucial for this regulation to occur: these molecules appear as a catalyser for the remodeling of the actin cytoskeleton underneath cell-cell contact, enabling a differential contractility between the cell-medium and cell-cell interface to take place.

## Introduction

Thanks to the pioneer's work of M. Steinberg, tissue surface tension (TST) has appeared as a very robust tool to predict tissue envelopment behavior [1]. TST reflects intercellular cohesion, it is an equilibrium quantity and has the unique property to predict cell rearrangement in tissues. How to measure this very interesting and powerful quantity? Surface tension can be envisioned either as the difference of free energy between two states (attached and not attached) or as the work necessary to separate two surfaces initially in contact. Designing an experiment for trying to apply the second definition is unfortunately impossible. As if one would want to calculate surface tension by applying a force to separate the two surfaces (for calculating a work), one should do that in a quasi-static way, which on biological systems is clearly impossible. So, one should not interpret experiments on the pulling of cell doublets - through AFM cantilever or by aspiration- as experiments giving access to surface tension. In this case, people are quantifying what is often called as the “strength of the contact” but which is an undefined mixture between equilibrium and dynamical quantities. Indeed, results are clearly influenced by the pulling rate as the remodeling of the contact zone while stretching can be very important. Thus, as pointed out by Steinberg, the measurement of surface tension with a tensiometer as developed for the first time by Foty [2] and as reimplemented here by the authors is one of the only ways to get access to this equilibrium quantity. In this sense, surface tension is much easier to understand as a difference in free energy between two states attached or not attached.

However, the microscopic origin of this surface tension is not clear. Steinberg proposed the Differential Adhesion Hypothesis (DAH) to explain the origin of surface tension and the variation from tissue to tissue. He gave a predominant role to the adhesion energy due to surface adhesion proteins [3], such as cadherins [4], and postulated that surface tension was directly a measurement of the cadherin-cadherin interactions. From expressing different levels of cadherins in cells, Foty and coworkers got a linear relationship between the cadherin expression level and the surface tension [5]. However, using the average bond energies of cadherin pairs measured by surface force apparatus [6] and the various estimates of cadherin density using different methods (*i.e*. 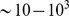 bonds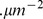) [5], [7], [8], we get two to three orders of magnitude lower surface energies than measured TST [5]. Different complementary experimental approaches suggest that cell sorting *in vivo* and *in vitro* is not governed solely by protein-level differences in cadherin adhesion [6], [9], [10]. But already in 1976, Harris pointed out that surface tension may have different origins. He, in particular, proposed a differential contraction hypothesis [11]. This idea will be later used for computer simulations of cell sorting [12]. And recent studies have indeed highlighted the important role of the contractile actin system in the organization of tissues [12]–[14]. In particular, Maître *et al*. calculated the ratio of cortical tension to adhesion energy and showed that the latter is considerably lower than cell-cell cortical tension. In the light of these findings, one can question again Foty's experiments and ask whether modifying the expression level of cadherins was not also modifying the contractility at the cell-cell, cell-medium interfaces. One of the goals of our study is to have a better understanding on the respective roles of adhesion and contractility on tissue surface tension and to estimate separately adhesion energy from cortical tensions.

Moreover, pursuing this fluid analogy, it appears that another parameter is of great importance if we want to understand the dynamics of tissue reorganization: the tissue viscosity 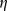, as the TST 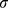 does not tell us anything about the dynamics. Different techniques were proposed to measure this parameter. Forgacs *et al*. interpreted their force relaxation behavior in a tissue surface tension measurement as a viscoelastic relaxation from where they could deduce a viscous parameter [15]. Others analyzed the kinetics of fusion of two aggregates [16]–[18] in order to measure the visco-capillary velocity 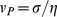. Recently, relative viscosity values were measured in two different geometries by Brochard and coworkers, namely the aspiration of aggregates in capillary tubes [19], and the spreading of aggregates on adhesive substrates [20]. Gathering data obtained from these different studies, we can note a slight correlation between viscosity and TST with a range of viscosity values between 0.2 and 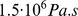. However, no systematic measurements were performed as a function of cadherin expression or cell contractility, and that is what we tested here (for the contractility dependence) using the fusion assay experiment.

If we want to understand the dependence of surface tension or viscosity on the underlying properties of the actin network, we really need a comprehensive description of the very dynamic and very complex interplay between the cadherins and the cytoskeleton. Many reviews have been written around these issues recently [21]–[24]. Let's put here a special emphasis on one of the key partner in the adhesive complex: 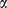-catenin. This protein has long been thought as the one protein which mediated the link between cadherin and the actin cytoskeleton. However, later on *in vitro*, it was shown that 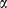-catenin could indeed bind either to the 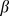-catenin-cadherin complex or to actin, but not to both at the same time [25]. If the link has to be done, we will have to find another protein. EPLIN might play this role [26]. To make the scene even more complicated, it appeared recently that 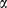-catenin was also a tension transducer [27] and was able to change conformation under tension and to recruit vinculin while stretched at the adhesion zone [28]. But if one wants to really understand the cadherin-actin complex, it is not just the eventual physical link which has to be considered but much more the dynamics of the reorganization of the entire network underneath the contact zone which occurs during the formation of cell-cell contact. Yamada and Nelson have thus studied the growing E-cadherin contacts between MDCK cells [29] and observed a complete remodeling of the actin network at the first stage of contact formation: the actin cortex bundle which normally surrounds the cell is literally dissolved leaving actin bundles bracketing the expanding edge contact. If myosin was reported to be present at cell-cell junctions [30], activated (phosphorylated) myosin is only found on cell periphery [29]. What do we know about the regulation of this remodeling? Formin has been shown to be associated with 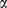-catenin [31], and so it is envisioned that it favors the apparition of actin bundles, while Arp2/3 was shown to be activated on the side of the cadherin-adhesion zone, favoring the growing of the contact (for a review, see [22]). We will show here that not only 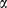-catenin is essential for the remodeling to occur but moreover that it is an essential element for the regulation through contractility of tissue's essential parameters that are viscosity and surface tension.

To do so, we used F9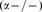 cells which have been knocked out for 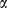-catenin. This loss does not change the level of cadherin expressed by F9 cells [32]. To get further information on the role of contractility, we also investigated and quantified the effects of three different drugs, with opposed actions on the cytoskeleton: nocodazole, Y-27632 and blebbistatin. The first one, nocodazole, is expected to indirectly increase stress fiber contractility by inhibiting microtubule dynamics and activating the rho kinase pathway [33]. The enhancement of cell contractility is achieved in a cadherin independent manner. In opposition to nocodazole, the second drug we exposed our aggregates to, Y-27632 dihydrochloride monohydrate is known as a selective inhibitor of Rho-associated protein kinases (ROCK-I and ROCK-II), thus implies a decrease in cell contractility [34]. Finally, the third drug we used, blebbistatin, is a selective inhibitor of non-muscle myosin II [35].

Combining these two approaches and adding to them angle measurements has enabled us to measure quantitatively the respective role of adhesion and contractility on tissue surface tension and viscosity and to highlight the essential role of 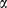-catenin.

## Results and Discussion

### Evaluation of cortical contraction and adhesion energy: model and assumptions

We will first present the framework used for interpretation of our data, as it enables understanding the choice of the different experimental conditions. We used the Differential Interfacial Tension Hypothesis (DITH) theory, which has been introduced several years ago now in order to accommodate for both the role of adhesion and contractility [17], [18], [36]. It relates TST to the tension along the edges of individual cells. The surface tension is a differential in interfacial energy, whatever the origin. So, one can assign an energy to the interface of the cell, which we could write [37]:
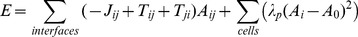
(1)


**Figure 1:**
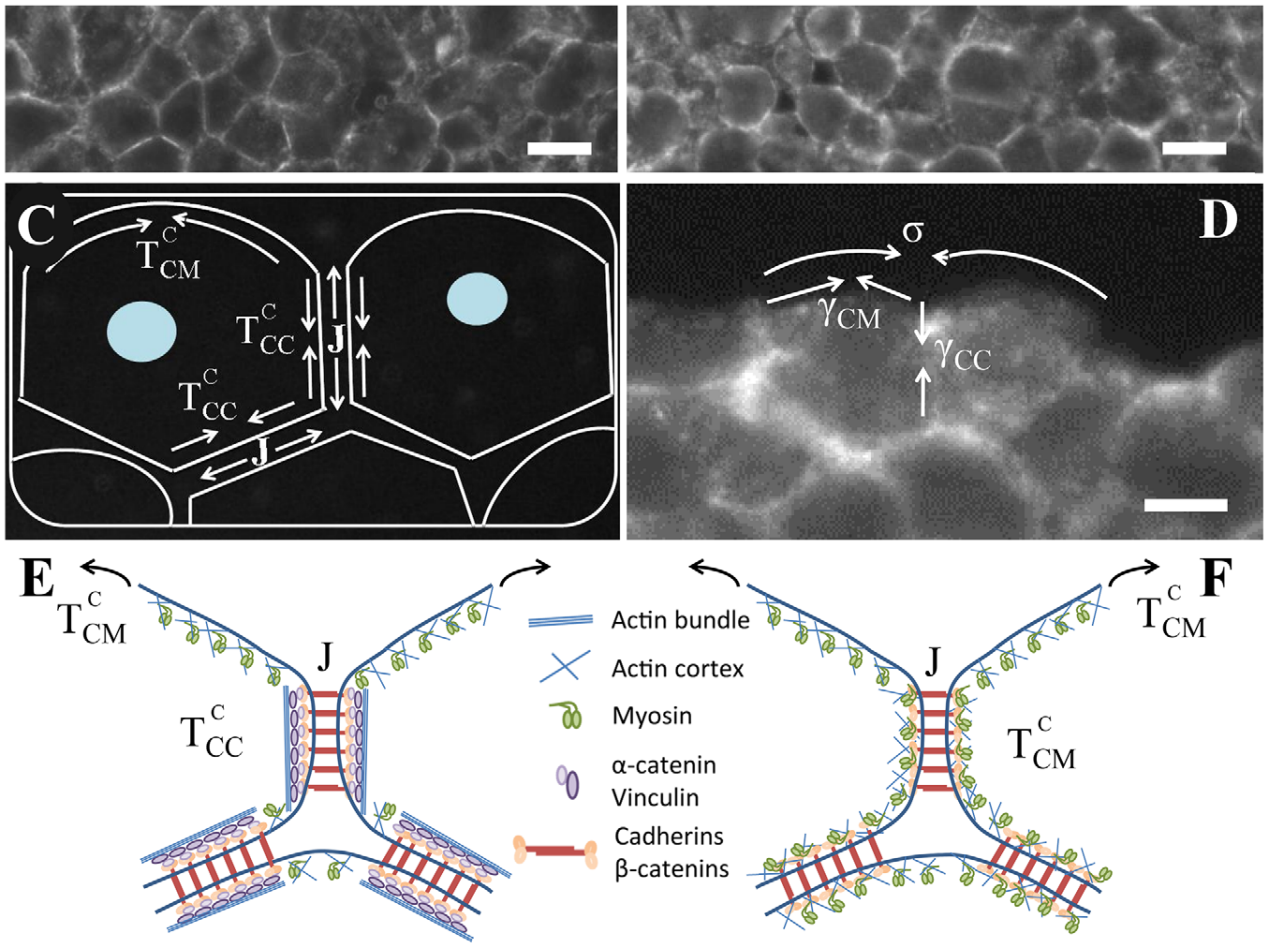
F9 versus F9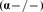. Cross sections of a F9 WT aggregate (A) and of a F9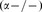 aggregate (B). The staining is for 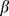-catenin, scale bars, 10 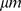. (C) Schematic representation of the cortical contractions at the cell-extracellular medium interface (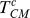), at the cell-cell interface (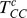) and of the adhesion energy J. 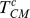 and 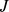 increase the tissue surface tension, whereas 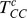 decreases it (see Eq. 2). (D) Closer view of two F9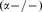 cells in contact. The difference between the interfacial tensions at the cell-cell 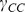 and cell-extracellular medium 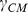 interfaces gives the aggregate surface tension (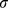). 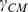 increases the surface tension whereas 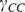 decreases it. Scale bar, 5 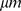. (E-F) Cartoons of cell-cell contacts in the case of F9 WT cells (E) and F9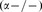 cells (F). The presence of 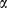-catenin at cell-cell junction is described as a catalyzer for actin reorganization at cell-cell junctions.



The definition of the different parameters is exemplified on Fig. 1E–F. For aggregates with only one cell type, 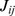 is the adhesion energy in the sense of Steinberg between cells which is taken positive (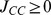) or between a cell and external medium 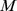 which is taken as the reference energy (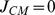). 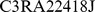 are the cortical line tensions along the side between cells or cell and medium. 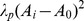 corresponds to an elastic cortex term (here written uniform for all cells). The interfacial tension 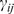 is the energy needed to increase an interface by one unit. Mathematically, this will write down as: 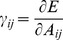. Hence, 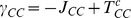 and 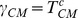 where 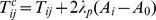 is an effective cortical contraction. One can even refine the model and have 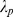 depending on the contact line. In the followings, we will not discuss these issues, we will not be able even to tell what is the prominent part of the cortical contraction 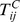: the line tension term (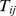) or the elastic term proportional to the perimeter. This point is discussed in other studies [38]. The TST 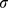 is defined as the difference in free energy between a state where two cells are in contact with each other and a state where the same cells are separated apart far from each other.

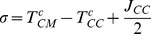
(2)If contractility is the motor of surface tension, it immediately implies that the contractility at the cell medium interface is higher than the one at the cell-cell interface.

The origin of 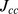 is now discussed. As stated in the introduction, as an equilibrium quantity, it is only linked to the difference in intermolecular interactions at the surfaces and again, no dynamical considerations or remodelling should be taken into consideration here. Now we can envision two different scenarios:

The molecular interaction between two cells is dominated by the interaction between cadherins (as proposed by M. Steinberg a while ago) and as long as the number of cadherins (whatever the distribution) is the same on cell surfaces the adhesion energy can be considered as constant in the different conditions.
The molecular interaction between two cells is highly nonspecific and the adhesion energy will clearly be linked to the average distance in between cells (that is where cadherins play a role by making it possible for membranes to be in close contact, and to maintain a controlled distance in between the membranes). So as long as their numbers stay roughly the same at the surface, the adhesion energy can be considered constant in between the different conditions.

Whatever the real physical origin of this energy, it will always be noted 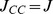 thereafter. In the following, we will use 2 different cell types F9 WT and F9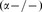 cells, for which it was shown that the number of cadherins expressed at the surface stays the same [32]. In the same way, we used different drugs for which we knew that the number of cadherins expressed at the surfaces stay the same: blebbistatin and Y-27632 [39], and also nocodazole [40]. Note also that the distribution of cadherins stays homogeneous as shown by the 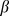-catenin staining in the case of F9
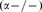
(compare Fig. 1 A and B). That is why we make the first hypothesis that for all these conditions the adhesive term J remains the same. We will see that this is very robust to the interpretation of our data. The next assumptions used to interpret the TST measurements are related to the relative values of cortical tensions. The membrane of a cell with no contacts is surrounded by a cortical mesh which is contracted by myosin activity. What do happen when a contact is being formed? That is what is schemed on Fig. 1 E–F. When 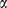-catenin is present, we postulate that the entangled cortex mesh is completely remodeled to form actin bundle whose contractility can be regulated, whereas when 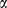-catenin is absent the meshwork stays more or less as it was before the initiation of the contact. 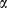-catenin appears as a key regulator which enables the complete remodeling of the cortex underlying the adhesive contact zone. Without it the cortex remains uniform in the cell and 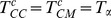. This hypothesis is also supported by the histological cuts presented in Fig. 1 A–B. The cells in a F9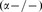 aggregate (B), appear much more rounded (uniform tension on every side, no remodeling) compared to a F9 aggregate where some remodeling seems to have occur as the contact zones can be very well assimilated to straight lines. As a consequence, the surface tension is given only by the adhesion energy 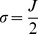.

For the F9 WT cell aggregates exposed to 10 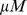 Y-27632, or 10 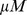 blebbistatin, myosin II is inactivated, therefore the cell contractility is largely but not totally removed [41]. The mechanisms of the myosin II independant (MI) tension regulation are still largely unknown but one can assume that cortex remains mainly uniform after such a treatment and that again 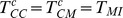, 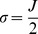.

### Quantitative dependence of TST on contractility

**Figure 2:**
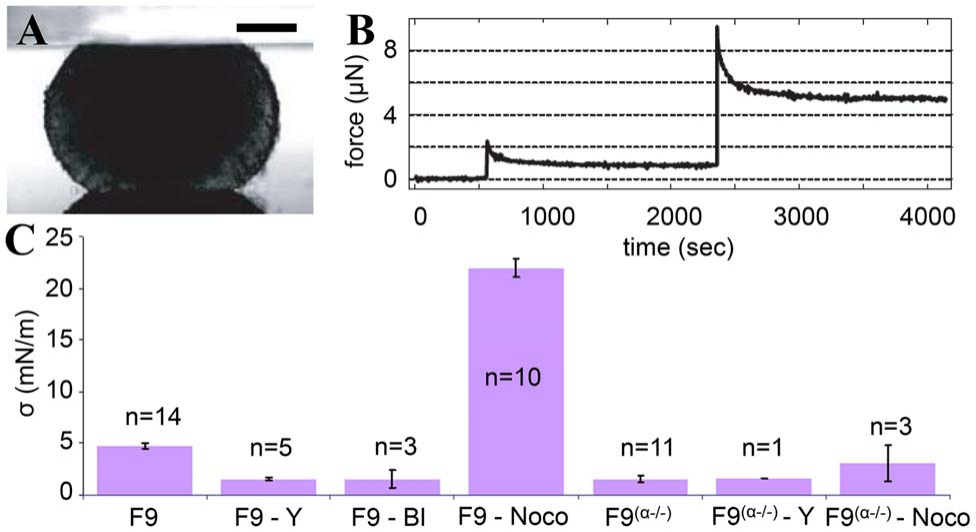
Quantitative dependence of TST on contractility. (A) Side view on a stereomicroscope of a F9 WT aggregate compressed between two parallel plates. Scale bar 150 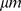. (B) Force signal obtained when subjecting a F9 WT aggregate to 2 successive compressions. (C) The value of the TST for different cell lines and drugs as obtained from tissue surface tensiometry experiments. Error bars represent standard errors on the mean (95 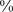 confidence interval of the mean) and n is the number of experiments, each experiment corresponding to several successive compressions.

We first began to measure the relative contribution of adhesion and contractility on surface tension. Few methods exist to measure TST: axisymmetric drop shape analysis of centrifuged aggregates [42] or aspiration of cell aggregates in a micropipette [19]. However, the most widely used quantitative method remains the compression plate tensiometry [2], [43]. It is based on the fact that, at long time scales, once elastic forces are relaxed, the surface tension (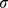) can be measured by assuming that cell aggregates verify the same physical laws of capillarity as liquid droplets. Thus, aggregate compression experiments on the tissue tensiometer allow us to estimate aggregate surface tension by recording the force signal when a plateau is reached and the shape parameters of aggregates (Fig. 2A–B). For the profile's analysis, we are using a local polynomial fit [44]. Surface tension is independent of the number and magnitude of compression up to 50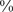 compression rate [44]. Moreover, it is also independent of the aggregate's volume (Fig. S1A) indicating that we are measuring a true surface tension. We obtained for the F9 wild type (F9 WT) a surface tension of 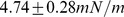 (n = 14 aggregates).

The surface tension for F9 WT cell aggregates decreases when exposed to 10 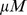 Y-27632 and to 10 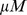 blebbistatin, down to 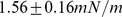 (n = 5 aggregates) and 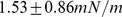 (n = 3 aggregates) respectively, as shown in Fig. 2C. As expected, the surface tension largely increases when F9 WT aggregates are treated with 1 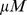 nocodazole up to 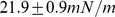 (n = 10 aggregates). In this case, equilibrium is not reached as the force is continuously decreasing even after two hours of relaxation. However, having done the analysis at different stages of the relaxation time, we can still state that the values for the surface tension are much larger than for non-treated aggregates. The TST for F9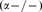 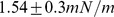 (n = 11 aggregates) is almost identical with the one for the WT treated with 10 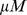 Y-27632 or with 10 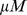 blebbistatin. Also, we have a slight increase of the surface tension for F9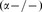 when we exposed them to 1 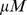 nocodazole (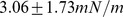, n = 3 aggregates). Preliminary tests with F9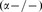 aggregates treated with 10 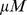 Y-27632 gave a surface tension of 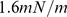 (n = 1 aggregate). All our results present quite small error bars because experimental dispersion is rather low. All differences with the parent cell line F9 are statistically significant, as determined by using Student's t-tests where 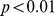. They come to confirm that surface tension strongly depends on cell contractility. From measurements on F9 WT, we have seen that the surface tension is highly dependent on the contractile behavior of cells, whereas in the case of F9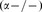, this dependence disappeared.

### Interpretation

Using the DITH and the above discussed assumptions, one can readily interpret our data. The fact that F9 WT cell aggregates exposed to 10 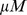 Y-27632, or 10 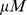 blebbistatin and F9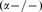 aggregates possess a same surface tension 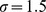 mN/m is coherent with our assumptions that nor the absence of 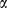-catenin neither the addition of the drug modify the value of the adhesion energy parameter 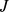 and that 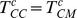 in all cases. From the expression 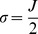, we obtain a value for 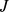 of the order of 3 mN/m. The model also allows us to compute for the F9 WT cells the difference between the two cortical contractions. We obtain a value of 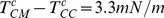, which means that 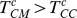. For our WT cell line, the difference in cortical tensions 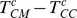 contributes to two thirds of the surface tension value and the adhesion 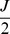 to one third only. Matre *et al*. found recently even a lower adhesion contribution [14]. As seen in the introduction, this is much lower than the value expected if we only take into account the trans-interaction between the extracellular domain of cadherin. However, one can expect that computing adhesion between the two surfaces may not be simply due to the trans-interaction between single cadherin, there will also be lateral specific or unspecific interactions. Moreover, as cadherins are not completely recovering the adhesion zone but often form patches, one may have to add unspecific adhesion between the rest of the membranes in between these cadherin clusters which may be high, as membranes seem there in close contact. It was suggested that cell/substrate adhesion of *Dictyostelium* cells migth be mediated by van der Waals attraction of their surface glycoproteins to the underlying substratum [45]. Such a unspecific adhesion mechanism with sugars migth take place for cell-cell contacts. Depletion forces might also play a role, as the contact zone distance will entail exclusion of a part of the big molecules that the medium may contain.

### Getting access to values of 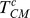, 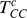 independently: angle measurement

If we assume a local mechanical equilibrium at the three phase cell/cell/medium contact line (origin of the angle 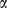 on Fig. 1C), the following relation should hold:
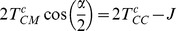
(3)


Hence, in principle by measuring the angle 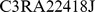 we have a supplementary equation that allows the determination of all effective cortical contraction parameters. The histological cuts allowed us to estimate the contact angle between cells for the two different cellular types. For F9 WT cell aggregates, we measured an angle 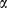 (see Fig. 1 C) of 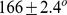 (n = 11), while for the F9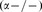 cell aggregates this angle becomes smaller 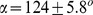 (n = 17).

We completed these measurements with the analysis of aggregates' rugosity. (Fig. S3 shows F9 WT and F9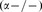 cell aggregates after two days in hanging drops and another two days on the gyratory shaker. We can see that it is possible to just evaluate by eye the two images to state that F9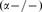 cell aggregates present a more accentuated profile rugosity. Using exponential curve fitting we measured for the F9 WT a value of 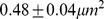 (n = 30 areas) and for F9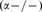 the value of 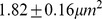 (n = 16 areas). These roughness values are compatible with the angle measurements: *i.e*., the smoother F9 WT aggregates present angles close to 180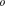.

If we reinject Eq. 3 in 2, we obtain: 
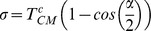
 We then obtain that 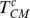 for F9 WT is equal to about 4.5 mN/m and 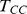 is equal to about 1.5 mN/m, whereas for F9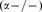, 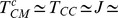
3 mN/m. It is not surprising here to obtain a different and lower value of 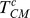 in the case of the mutant as it has been shown that depleting 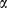-catenin often also induce a change in the ultrastructure of actin [46].

In conclusion, surface tension measurement combined to geometrical analysis appear to be a really powerful tool as to obtain all the desired tension parameters characterizing a cell line.

### Contractility dependent TST still predicts sorting out and envelopment

**Figure 3:**
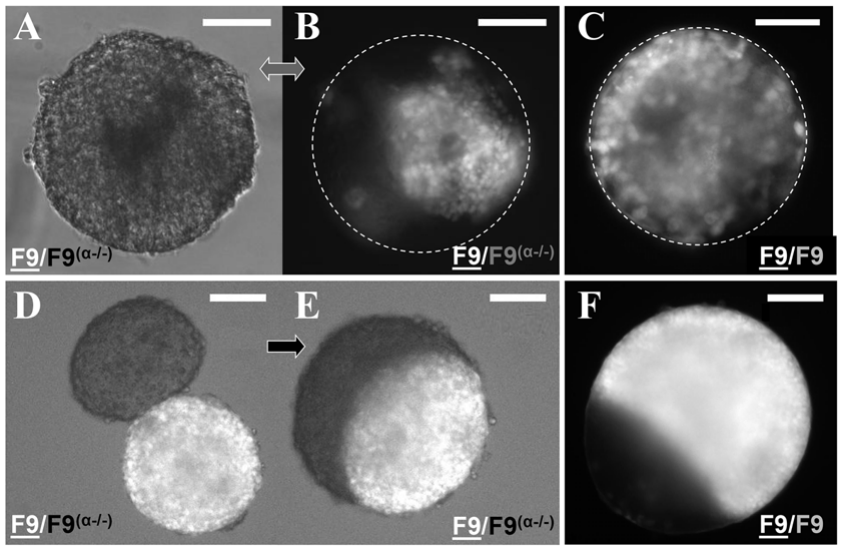
Contractility dependent TST still predicts sorting out and envelopment. Phase contrast and fluorescence images representing configurations at different stages of the sorting out (A–C) and envelopment (D–F) processes. (A–B) Phase contrast and fluorescence images correspond to 72 hours after mixing F9 and F9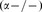 dissociated cells in hanging drops. (C) Control fluorescence of a F9/F9 mixture after 72 hours. (D–E) Engulfment of a F9/F9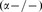 pair of aggregates 3 hours (D) and 29 hours (E) after the aggregates were brought in contact and allowed to fuse. (F) Final configuration (72 hours) of a F9/F9 pair of aggregates (control). Left right thick double arrow indicates simultaneous recording in phase and fluorescence. Rightwards thick arrow indicates evolution with time. Scale bars, 100
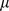
m.

We performed segregation assays in order to test whether the relative spatial positions adopted by two distinct adhesive cell populations which are mixed together (sorting-out) or by two separate aggregates brought in contact (envelopment) correlate with their surface tension. These phenomena are reminiscent of viscous liquids. The DAH predicts that in the case of two different tissues, the one of a higher surface tension should be surrounded by the less cohesive one, which has a lower surface tension [1]. We tested the ability of two distinct cell populations to sort out according to their surface tension, by generating aggregates using mixed suspensions of fluorescent F9 (named F9) and F9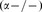 cell lines. Easily evaluated by eye after several hours, the sorting-out process showed that F9 cells tend to adopt an internal position while F9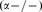 cells fill the external space of the newly formed aggregate (Fig. 3A–B). Following the same concept, we combined F9 and F9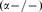 already formed spherical aggregates as presented in Fig. 3D. After 29 hours in hanging drop culture at 37°C and 5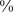 CO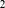 we evaluate the envelopment and we state that the F9 aggregate is completely surrounded by the F9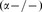 one forming a partial “sphere within a sphere” configuration (Fig. 3E). For both segregation assays, control experiments were made using a single cell line (*i.e*. F9 and GFP labeled F9 cells). No sorting could be detected, as even after several hours following the formation of the aggregate, the cells still present a mixed configuration (Fig. 3C). In the envelopment assays, no difference in surface tension was detectable as none of the cell line was enveloping the other (Fig. 3F). This certifies that the GFP labelled F9 cells present the same physical properties as the F9 line and didn't suffer any changes in terms of surface tension, following their transfection.

### Tissue fluidity versus contractility

**Figure 4:**
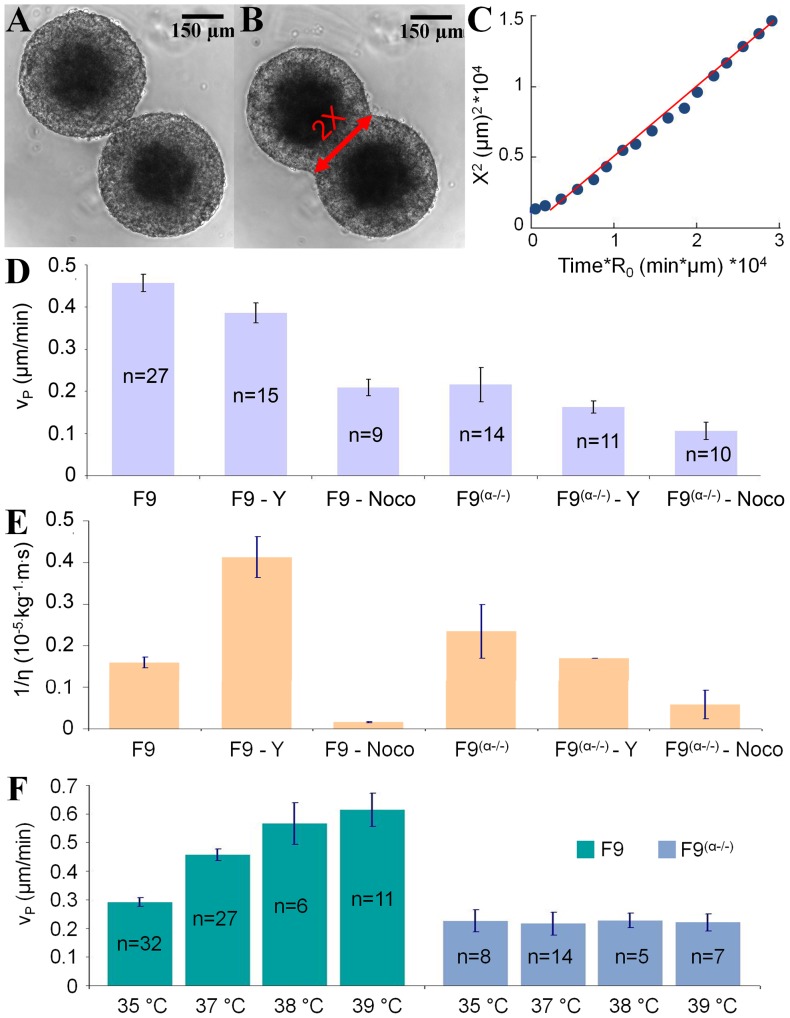
Tissue fluidity versus contractility. (A–B) Images of two fusing F9 WT aggregates corresponding to the beginning and the end of the analyzed period, showing in red the neck's diameter 2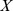. (C) Plot of 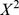 versus 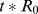 (time 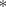 aggregate initial radius). Blue points represent experimental data and red line represents linear fit with the slope defining the visco-capillary velocity 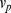. (D) Measurements of 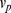 for different cell lines and drugs. Error bars represent the standard errors of the mean (95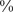 confidence interval of the mean) and n is the number of experiments. (E) Fluidity deduced from 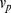 and 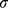 (*i.e*. defined as 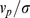) for untreated and treated aggregates and interpreted as the inverse of the viscosity. (F) Measurements of 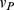 as a function of temperature for F9 WT and F9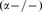 aggregates. Error bars represent the standard errors of the mean (95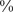 confidence interval of the mean) and n is the number of experiments.

The sorting out, rounding up and fusion of cell aggregates processes are driven by the tissue surface tension 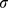 and resisted by the tissue viscosity 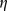. The fusion assay offers a quick and convenient way to estimate viscous forces (Fig. 4 A and B). The time dependence of the square of the radius neck 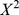 is linear at short times (Fig. 4C) as expected from the Frenkel's modified equation (see Materials and Methods) valid for purely viscous fluids (Newtonian). This equation gives us an estimation of the visco-capillary velocity 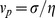.

Fig. 4D summarizes all the results on our two cell lines treated or not with contractile drugs. The visco-capillary velocity of untreated F9 aggregates remains constant at 0.46
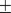
0.02 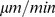 (n = 27 pairs) over more than a 3-fold range of diameters (Fig. S1B). For F9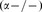, 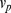 is approximately twice smaller at 0.22
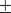
0.04 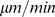 (n = 14 pairs). The use of 10 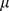
M Y-27632 generates a slight decrease in 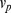 for both cellular types: 0.39
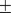
0.02 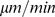 (n = 15 pairs) for F9 WT and 0.16
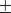
0.01 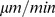 (n = 11 pairs) for F9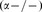. Aggregates treated with 1 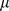
M nocodazole displayed a lower 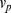 than untreated ones: 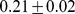 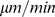 (n = 9 pairs) for F9 WT and 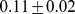 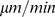 (n = 10 pairs) for F9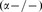. One striking feature emerging from this graph is the fact that the highest speed is obtained for the wild type condition, and whatever the drugs or genetic modification targeting the cytoskeleton contractility does not accelerate the process as if in the native condition a kind of optimization was attained.

Using the mean TST experimental values of Fig. 2, we can calculate tissue viscosity or tissue fluidity 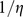 which is just the inverse of viscosity (Fig. 4E). For untreated F9 cell line, we obtain a high viscosity value of 6.2 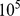 Pa.s in the range of values obtained in the literature [16]–[19]. Adding Y-27632 on the F9 cell line significantly increases fluidity. The absence of 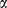-catenin as well increases slightly but significantly the fluidity as compared to the parent cell line. The difference is not significant between F9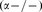 cells treated or not with Y-27632. The results obtained with nocodazole are consistent with the global tendency that fluidity is negatively correlated with cell contractility: fluidity is always lowered by adding nocodazole which increases contractility both in F9 and F9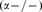 cell lines.

Finally, it is interesting to consider the effect of temperature on 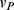 (see Fig. 4F and Fig. S2A–B). Temperature 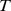 has a strong effect as 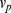 increases almost linearly with 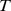 with a 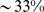 increase per degree. This indicates that as expected the viscosity and surface tension are regulated by active biological processes. On the other hand, the F9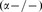 cell line does not present any temperature dependence, as if 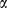-catenin was essential for these active processes to occur.

### Conclusion and Perspectives

By combining TST measurements and modification of contractile properties of cells, we have seen that our data were fully compatible with the DITH framework. Using this model, we can get a measurement of 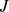 and 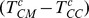. Angle measurements enabled us to get access to values of 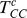 and 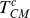 independently. Supplementary experiments through AFM or micropipette aspiration may give access to 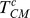 and thus enable a validation of the methodology presented here. We have also seen that both surface tension and fluidity were very dependent on contractility, and that 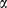-catenin was essential for this fine tuning of the macroscopic parameters. In the same order of idea, we have seen that the ratio 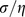 is very dependent on temperature for F9 WT, whereas the one of F9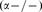 cells is not which means that, 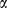-catenin is essential for a spreading of cells upon one another through active processes, and removing this essential protein lead to a passive spreading through physical interaction (most certainly non specific 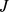). We can imagine how important it is during morphogenesis for the organization of tissues to have a mean to finely tune the fluidity of one tissue relatively to its neighbor. It appears here that the subtle reorganization of the actin network triggered by 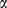-catenin gives the tissue such a possibility simply by regulating its contractile activity.

## Materials and Methods

### Lines and Aggregates Preparation

Mouse embryonic carcinoma F9 cell line and its derivatives were a generous gift from A. Nagafuchi (Kumamoto University, Japan) [47]. For the cell sorting-out and envelopment studies, we used F9 cells labeled with Histon-GFP (referred to as F9) [48]. Histone GFP DNA was again a generous gift from A. Nagafuchi. Details on this vector can be found in [48]. For the transfection step, we followed the same protocol as described in [48]; expression vectors were transfected using the lipofectamine 2000 system (GIBCO). We used a 24-well culture plate for transfection. After one day the transfected cells were transferred into culture dishes. After two days culture medium was exchanged for one containing antibiotics (400 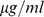 G418) for selection. Culture medium was changed every day for a week. And then, medium change was done every two or three days. Two or three weeks after transfection, we were able to isolate colonies. As previously described [18], cells were plated on plastic tissue culture dishes pre-coated for 15 min with gelatin (2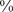, Sigma G1393) diluted to 0.2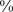 in PBS and incubated at 37°C and 5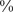 CO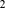 in DMEM (41965-039; GIBCO) supplemented with 10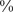 fetal bovine serum (PANSera ES, Dutscher 500105ES) and 1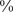 antibiotics (penicillin streptomycin, GIBCO Invitrogen corporation, Cat. no. 15140–122). To generate spherical aggregates, cells were dissociated and reassembled in 15 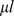 hanging drops [18]. After two days, the newly formed aggregates were transferred into fresh culture medium filled sterile non-treated plastic Petri dishes and then incubated on a gyratory shaker (at 160 rpm, 5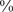 CO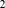, 37°C). Depending on the cellular type, the entire procedure took from three to five days and yielded spherical aggregates ranging between 180 and 500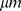 in diameter, each containing 1500 to 35000 cells. All experiments require for the cell aggregates to be transferred to CO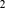 – independent medium (18045–054; GIBCO) supplemented with the same components as the culture medium. We investigated and quantified the effects of three different drugs, with opposed actions on the cell cytoskeleton: nocodazole (Sigma M1404), Y-27632 dihydrochloride monohydrate (Sigma Y0503) and blebbistatin (Sigma B0560). Aggregates were treated with 1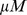 nocodazole, 10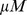 Y-27632, or 10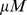 blebbistatin respectively. The drugs were added in the CO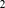 – independent medium only half an hour before the beginning of the surface tension and fusion experiments.

### The compression plate tensiometer

In order to measure the surface tension 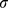 of our aggregates, compression experiments were done on a homemade surface tension apparatus [44]. As previously described [44], the aggregate, located between two parallel glass plates, is subjected to several successive compressions of steps going from 25 to 50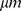. The two compression plates consist of a 2-mm thick borosilicate glass for the lower one (LCP) and of a cover glass surface for the upper one (UCP). A tungsten or inox wire, with diameter 0.1 and 0.8 mm respectively, connects the UPC to a copper-beryllium cantilever which has a spring constant varying between 0.36 and 0.67 N/m). The cantilever deflection is measured with a non-contact eddy current displacement and position measurement sensor (DT 3701-U1-A-C3, micro-epsilon). A NewStep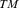 Motion Control System (NewPort) controls the z direction of the LCP through the movement with a controlled velocity of a cylindrical rod that traverses a CO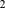 -independent filled chamber in which aggregates are deposited. The same chamber can be moved in the x,y,z directions to center the sample on the optical axis by an electronic micromanipulator (MP285, Sutter Instrument). The whole setup is embedded in a thermally isolated chamber maintained at the desired temperature (i.e. 37°C) by a resistance traversed by a current that is modulated by a temperature controller (331; Lakeshore). Evaporation of the medium is prevented by covering the free-open surface with a thick mineral oil layer (Sigma 330779-1L). To ensure a minimal aggregate adhesiveness, surfaces are prepared and treated following the protocol described in [44]. The monitoring of aggregates' shape is done using a stereomicroscope (MZ16 binocular, Leica) and a camera (A686 M; Pixelink). We use a KL 1500 LCD cold light source (Leica) with “flexible tubes” to adjust the lightning. The whole setup is controlled with Labview (National Instruments), and image analysis is performed with Matlab (The MathWorks) and ImageJ (National Institutes of Health, Bethesda).

### Fusion of aggregates

The measurement of apparent viscosity requires the recording of the kinetics of two fusing aggregates. For this to be achieved, before each experiment, a 48 well plate is coated with 1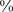 agar (SIGMA-A1296) prepared and deposited following the Sigma protocol in order to prevent adhesion of aggregates. Afterwards, the wells are filled with CO2 independent medium and according to the type of the experiment we can also add 1 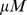 nocodazole or 10 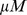 Y27632. A pair of aggregates, chosen as to have approximately the same diameter, is transferred in each well and we record, with periods from 5 to 10 minutes, the kinetics of the fussing process on a motorized Nikon Eclipse TE 2000 E microscope using NIS Elements. Experiments are carried out at thermal equilibrium, as the microscope is enclosed in a home-made polystyrene chamber, heated using a radiator controlled by a circulating water bath thermostated at 37°C. Image analysis (*i.e*., extraction of the neck's radius 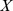) is performed with Matlab (The MathWorks) and ImageJ (National Institutes of Health, Bethesda). The initial regime of such fusion may be described by 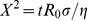 where 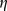 is the tissue viscosity, 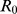 the initial radius of the aggregate, 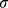 the tissue surface tension, and t the time. In the original work by Frenkel [49], there was a mistake about incompressibility, detected by Eshelby (page 806 of the discussion of [50]), who removed the extra prefactor 2/3 on the right-hand side. We also found a calculus error on the line above Frenkel's equation (7) and removed an extra prefactor 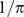 [18]. Our corrected formula therefore does not contain any numerical prefactor. This is consistent with a more extensive calculation of the fusion process at large times [17], [51].

### Cell sorting and tissue envelopment

For segregation assays we used the GFP labeled F9 cell type (F9), as it is suitable for visualization and evaluation by eye due to its fluorescence properties. For cell sorting assays, aggregates were prepared using a cell suspension containing two different cell types: F9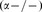 and F9 or the control F9 and F9. To evaluate whether the cell sorting takes place we observed the newly formed aggregates on a Nikon Eclipse 2000 fluorescence microscope at different stages of the phenomenon. Right after preparation and in between the images taking, the aggregates are incubated at 37°C and 5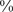 CO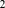. Spreading of one cellular type over another one was tested by using the same pairs of cells as for the sorting out assays, only this time, the interaction took place between already formed spherical aggregates containing only one type of dissociated cells. We tried two different methods to bring in contact the two distinct aggregates: placing them side-by-side on an agar bed or putting them together in a unique hanging drop. For both methods we monitored the combined aggregates every several hours.

### Protocol for cross-sections

For each cell type, a thousand of aggregates were collected in a 15 ml tube, centrifuged at 1200 rpm for 2 minutes and the supernatant was carefully removed. Next, they were fixed using 4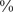 PFA (30 minutes), rinsed with PBS (4 times, 5 minutes with gentle agitation), dehydrated in a graded ethanol series (70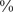, 95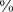, 100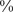, one hour each) and butanol (30 minutes) before embedding in paraffin/xylene (v/v) solution (60°C, over night). The aggregates were submitted to three steps of pure paraffin embedding (1 hour, 60°C) and air-dried. Sections (5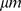 thick) were made using a microtome (Leica Microsystems) and transferred to glass slides.

### Immunohistochemistry

The aggregate sections were deparaffinized using xylene (15 minutes, twice), 100% and 95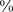 EtOH (10 minutes, twice) and rehydrated in PBS. The sections were incubated in PBS with 10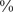 BSA for 30 min before incubation with antibody against 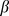-catenin (sc-7199, Santa Cruz Biotechnology, Inc.) at 1∶300 dilution (90 minutes, RT). The sections were then washed with PBS and incubated with Alexa Fluor 488 goat anti-rabbit IgG (Invitrogen Molecular Probes) at 1∶1000 dilution (1 hour, RT). Slides were mounted with fluorescent mounting medium and observed under a Nikon Eclipse 2000 fluorescence microscope.

### Profile rugosity measurements

We measured the rugosity of aggregates by analyzing their contour shape. F9 and F9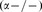 cell aggregates were prepared as presented above and after two days of gyration in the incubator we transferred them into CO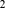 independent-medium filled culture dishes and we took phase contrast images on a motorized Nikon Eclipse TE 2000 E microscope using NIS Elements. Image analysis was performed with Matlab (The MathWorks). We convert our images to binary images, based on threshold. We use exponential curve fitting and we measure the areas given by the distance between the original contour and its curve fitting. To avoid areas in the contour where dead cells appear, we chose to analyze for the same aggregate several areas of different sizes. All analyzed images correspond to the same time in aggregate culture.

## Supporting Information

Figure S1

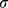
 **and 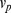 do not depend on aggregate's radius.** (A) F9 WT cell aggregate TST measurements as a function of aggregate radius. Each data point is the result of several compressions done on the same aggregate, and the value of the surface tension is the slope of the curve Force versus 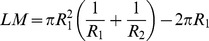, where 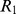, and 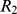 are the principal radii of curvature. TST values are independent of the aggregate size (radius). The error bars are given by the 95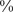 confidence interval given on the linear fit of Force Vs 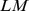 data. (B) F9 WT cell aggregate visco-capillary velocity 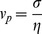 measurements as a function of aggregate radius. 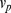 values are independent of the aggregate size (radius).(TIF)

Figure S2

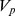
 **depends on temperature and drug treatment.** Visco-capillary velocity 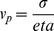 measurements as a function of temperature for F9 WT (A) and F9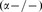 (B) cell aggregates untreated or exposed to 10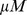 Y-27632 and 1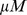 Nocodazole. Error bars represent the standard errors of the mean (95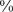 confidence interval of the mean) and n is the number of experiments.(TIF)

Figure S3
**Aggregate's rugosity.** Optical microscopy images of F9 WT (A) and F9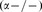 (B) cell aggregates of same age which serve for the estimation of the profiles' rugosity. The outlines prove that there is an evident difference between the two cell lines.(TIF)
