# Supplementary figures and images for: Fine Tuning of Tissues' Viscosity and Surface Tension through Contractility Suggests a New Role for α-Catenin
Source: PLoS One. 2013 Feb 4;8(2):e52554. doi: 10.1371/journal.pone.0052554 (PMC3563668; doi:10.1371/journal.pone.0052554)

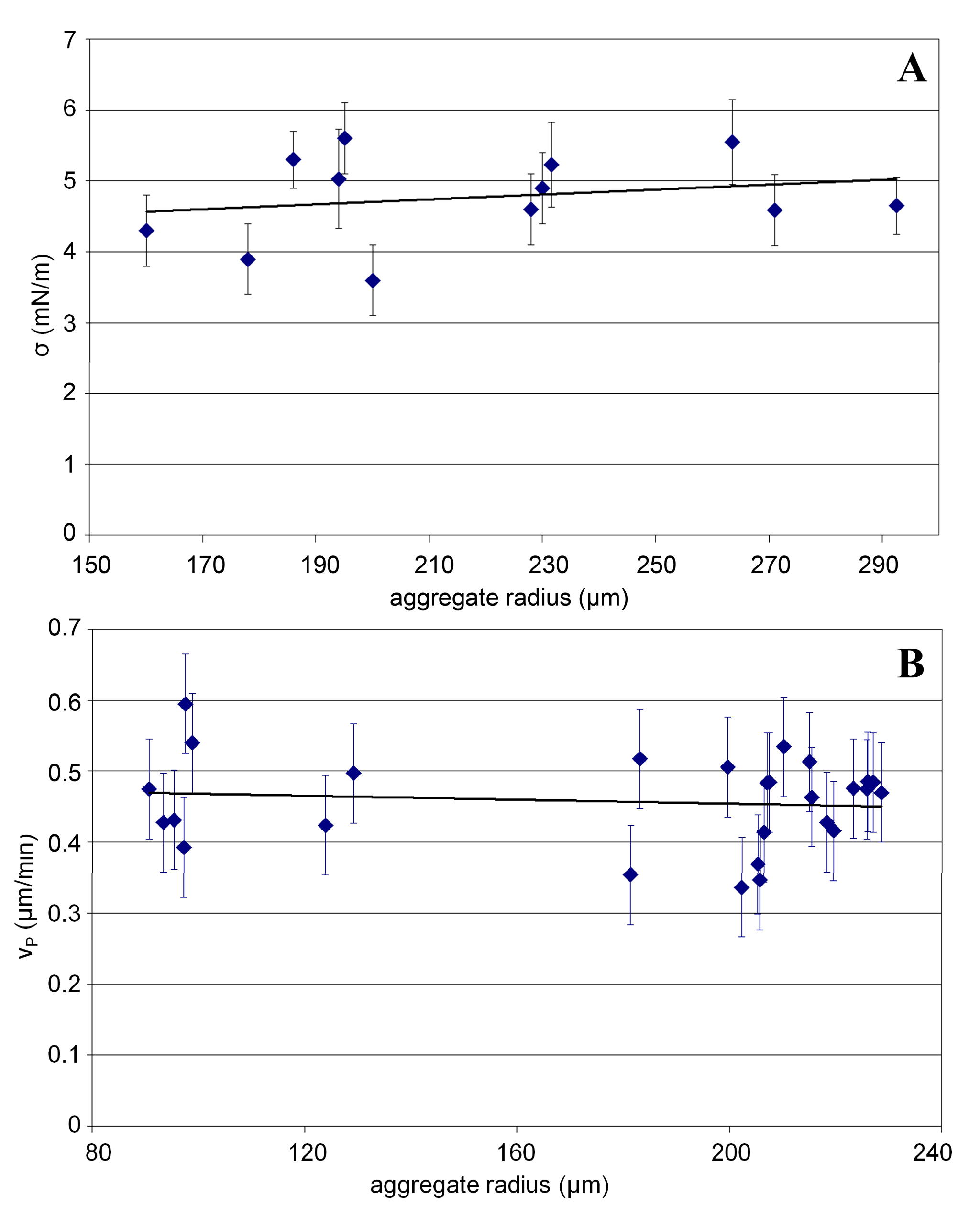

Supplement: Figure S1 — and do not depend on aggregate's radius. (A) F9 WT cell aggregate TST measurements as a function of aggregate radius. Each data point is the result of several compressions done on the same aggregate, and the value of the surface tension is the slope of the curve Force versus , where , and are the principal radii of curvature. TST values are independent of the aggregate size (radius). The error bars are given by the 95 confidence interval given on the linear fit of Force Vs data. (B) F9 WT cell aggregate visco-capillary velocity measurements as a function of aggregate radius. values are independent of the aggregate size (radius). (TIF) [file pone.0052554.s001.tif]

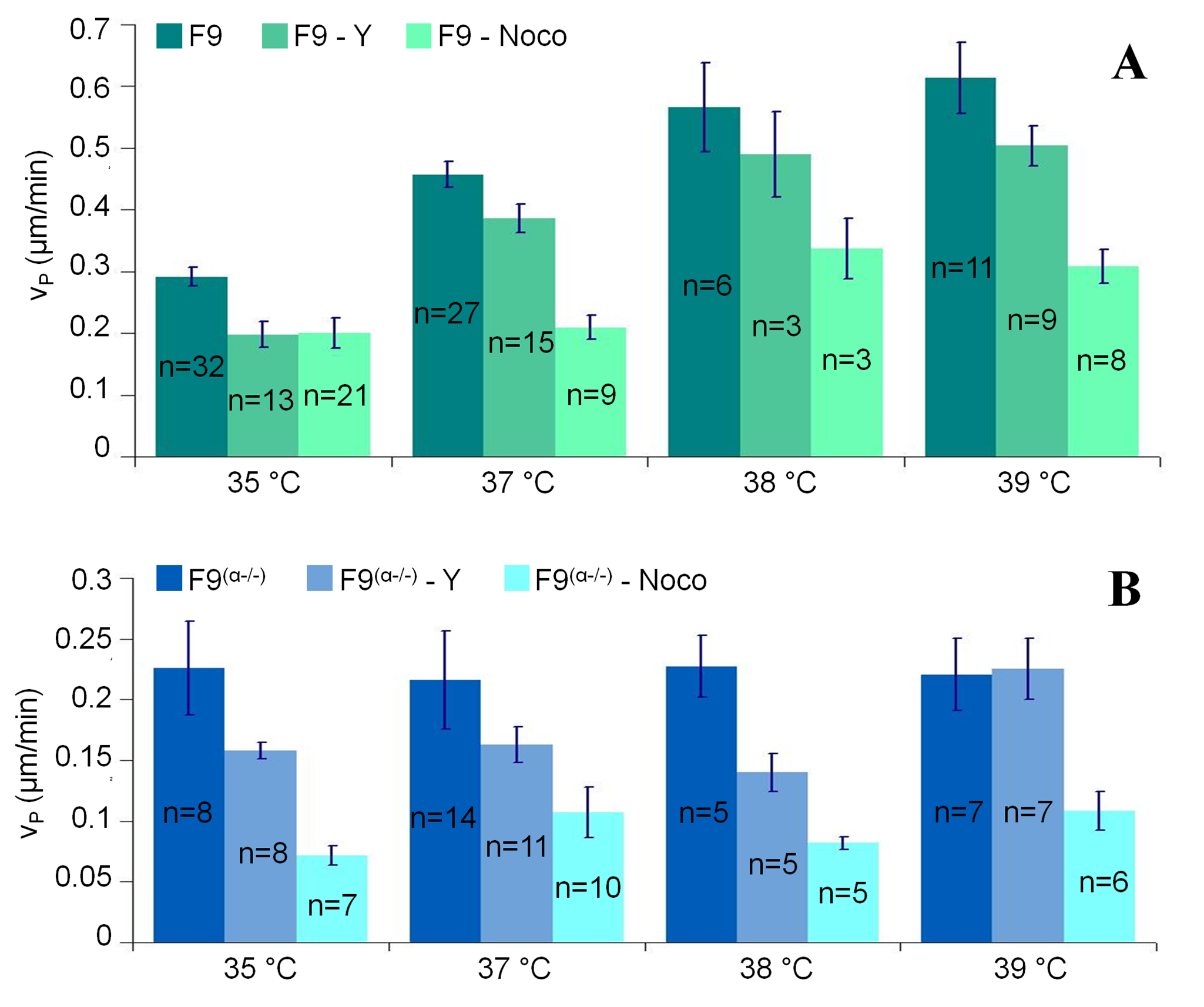

Supplement: Figure S2 — depends on temperature and drug treatment. Visco-capillary velocity measurements as a function of temperature for F9 WT (A) and F9 (B) cell aggregates untreated or exposed to 10 Y-27632 and 1 Nocodazole. Error bars represent the standard errors of the mean (95 confidence interval of the mean) and n is the number of experiments. (TIF) [file pone.0052554.s002.tif]

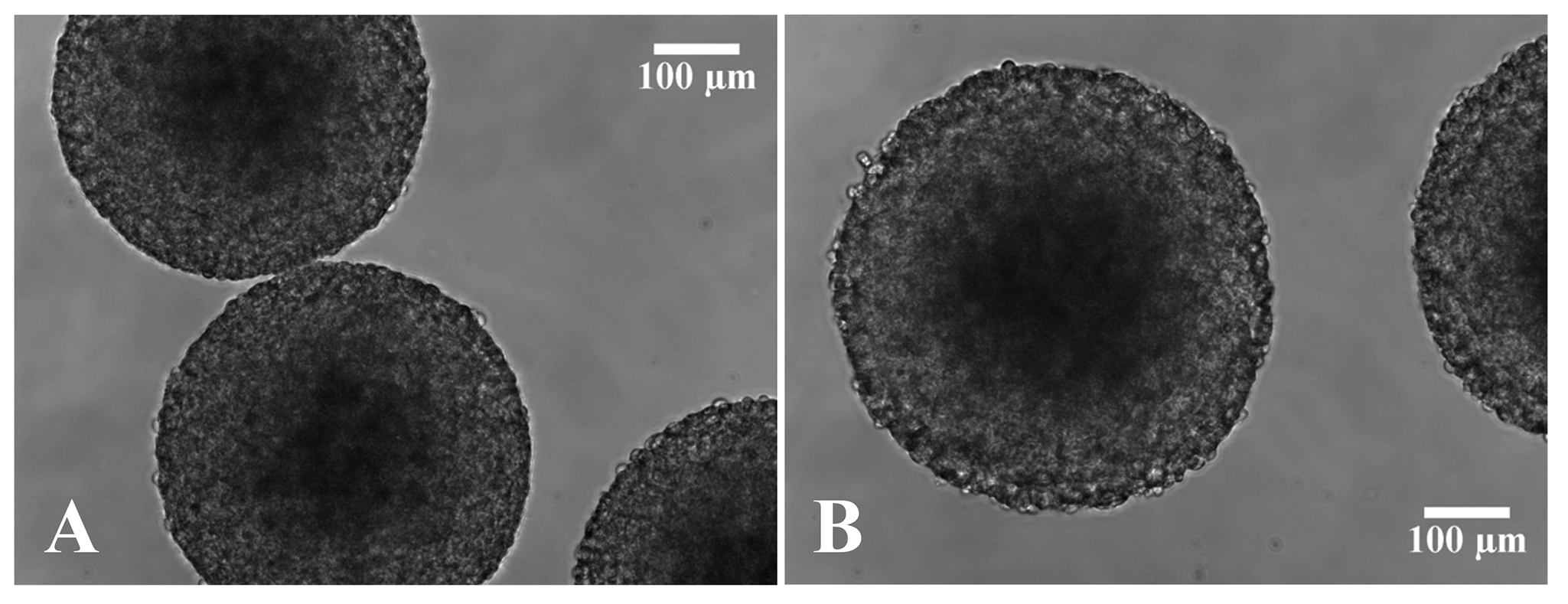

Supplement: Figure S3 — Aggregate's rugosity. Optical microscopy images of F9 WT (A) and F9 (B) cell aggregates of same age which serve for the estimation of the profiles' rugosity. The outlines prove that there is an evident difference between the two cell lines. (TIF) [file pone.0052554.s003.tif]
